# Persistence and Dropout in Higher Online Education: Review and Categorization of Factors

**DOI:** 10.3389/fpsyg.2022.902070

**Published:** 2022-05-31

**Authors:** Umair Uddin Shaikh, Zaheeruddin Asif

**Affiliations:** School of Mathematics and Computer Science, Institute of Business Administration, Karachi, Pakistan

**Keywords:** retention, persistence, attrition, dropout, online learning

## Abstract

Online learning is becoming more popular with the maturity of social and educational technologies. In the COVID-19 era, it has become one of the most utilized ways to continue academic pursuits. Despite the ease and benefits offered by online classes, their completion rates are surprisingly low. Although several past studies focused on online dropout rates, institutions and course providers are still searching for a solution to this alarming problem. It is mainly because the previous studies have used divergent frameworks and approaches. Based on empirical research since 2001, this study presents a comprehensive review of factors by synthesizing them into a logically cohesive and integrative framework. Using different combinations of terms related to persistence and dropout, the authors explored various databases to form a pool of past research on the subject. This collection was also enhanced using the snowball approach. The authors only selected empirical, peer-reviewed, and contextually relevant studies, shortlisting them by reading through the abstracts. The Constant Comparative Method (CCM) seems ideal for this research. The authors employed axial coding to explore the relationships among factors, and selective coding helped identify the core categories. The categorical arrangement of factors will give researchers valuable insights into the combined effects of factors that impact persistence and dropout decisions. It will also direct future research to critically examine the relationships among factors and suggest improvements by validating them empirically. We anticipate that this research will enable future researchers to apply the results in different scenarios and contexts related to online learning.

## Introduction

**Figure 1:**
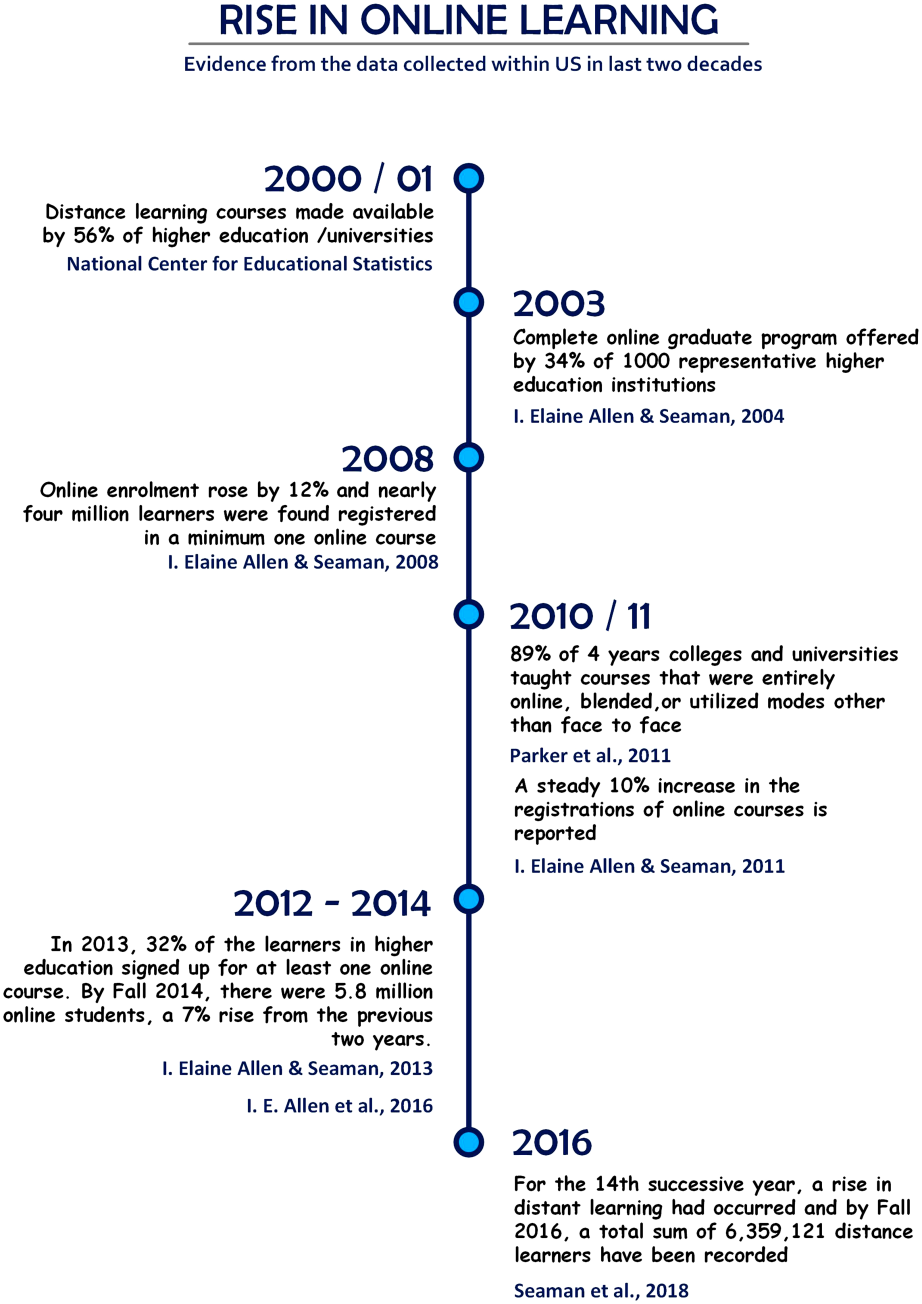
Rise in online learning in the United States.

Higher education is increasingly embracing online courses (Seaman et al., 2018; Johnson et al., 2019), mainly inspired by the demands of learners and budgetary constraints (Limperos et al., 2015). The popularity of online courses in the United States has increased significantly over the last two decades (see Figure 1), and there was a total of 6,359,121 distance learners as of Fall 2016 (Seaman et al., 2018). Similarly, more than 76% of colleges and universities in Canada offer online courses in 2019, and the proportion has risen to 92% of institutions with over 7,500 students and 93% of universities (Johnson et al., 2019). Online classes are considered effective as their face-to-face counterparts (Kumar et al., 2019). Students enroll in online courses to accomplish their own personal and professional goals. A greater degree of flexibility and unrestricted digital access to large volumes of information is compelling and accounts for the widespread popularity of enrolment in online courses (Sitzmann et al., 2006; Zimmerman, 2012). Accessibility to online courses empowers learners to structure their classes alongside other family and work commitments, which may not be possible otherwise (Lee, 2017). Also, the ongoing pandemic of COVID-19 has heavily impacted students, instructors, and educational organizations worldwide (Almanthari et al., 2020). The instructors moved their courses online, and the students remained at home in response to social distancing measures (Toquero, 2020). During these times, online learning became the most utilized way to continue academic activities globally, and experts began to consider it a viable alternative to face-to-face education (Kaur, 2020). Higher education institutes quickly adopted the online delivery of education, incorporating media and technology (Rahmat et al., 2022). They realized the need to develop and strengthen their capacity to achieve the desired results (Maqsood et al., 2021).

### Problem Statement

Despite the massive growth, persistence rates of online courses are significantly low (Xavier and Meneses, 2020) compared to those offered in person (Muljana and Luo, 2019; Delnoij et al., 2020). Online learners struggle to complete their courses (Friðriksdóttir, 2018) and attrition (or termination) is the leading problem encountered in many colleges (Bowden, 2008), which is a foremost challenge for online education administrators/instructors (Clay et al., 2008). The issue is still very challenging (Chiyaka et al., 2016; Hobson and Puruhito, 2018; Johnson et al., 2019; Li and Wong, 2019). Only about 15% of Open Universities students leave with degrees or other qualifications, indicating a meager persistence rate among students taking online courses (Mishra, 2017). Online dropout experience results in frustration and shatters learners’ confidence preventing future enrolments (Poellhuber et al., 2008), which implies inadequacy, questionable quality, and profit loss for institutions (Willging and Johnson, 2009; Gomez, 2013).

### Research Motivation

Many researchers realized the need to minimize dropout rates of online learners as beneficial for students, institutes, and companies over time (Lee and Choi, 2011; Wuellner, 2013; Garratt-Reed et al., 2016; Moore and Greenland, 2017; Murphy and Stewart, 2017). Additionally, the pandemic enforced utilization of technology in the learning process has made this vital topic of online learning more critical. Therefore, a need arises for further investigation into the quality of online learning (Basilaia and Kvavadze, 2020) from a new and improved perspective.

### Research Question

The decision to drop out does not always link to knowledge but may result from a lack of persistence. Persistence in online courses is considered a complex phenomenon influenced by many factors (Yang et al., 2017; Choi and Park, 2018). Any single factor cannot predict student attrition from online courses (Gaytan, 2013). It is imperative to study persistence on a large scale to understand better the factors that count toward online course completion or online learners’ decision to drop out (Choi and Park, 2018). The following research question guides the literature review based on the rationale provided.

What factors are positively or negatively linked with persistence in post-secondary online education settings?

### Persistence: Differing Definitions and Indicators

There is a problem with the non-standardized use of the term persistence in online courses. The authors either do not provide clear indicators for persistence or provide inconsistent definitions (Lee and Choi, 2011). Some authors have described persistence as an inclination to complete the currently enrolled online course (Joo et al., 2011; You, 2018), whereas others defined persistence as an intention to enroll in more online courses in the future or successfully concluding the course securing somewhere between A to C grade (Lee and Choi, 2011). Intention to persist in the currently enrolled online course is considered the most referenced indicator of persistence (Roland et al., 2018). We have relied on this exact definition in this study.

### Research Background

Several authors have studied persistence factors related to online courses in post-secondary educational settings (Gazza and Hunker, 2014; Muljana and Luo, 2019; Xavier and Meneses, 2020). These studies have used divergent approaches and frameworks, where authors have studied the factors in isolation. There exists a gap in the literature while analyzing the combined effect of factors on persistence and examining the impact of factors upon each other. To better understand the persistence or dropout phenomena, it is imperative to identify as many factors as possible and arrange them in their logical categories. In this study, we have reflected upon the factors that correlate positively (enablers) or negatively (barriers) to persistence in an integrative manner. This study contributes to the existing literature by presenting the organization of persistence/dropout factors, identified after a comprehensive literature review, as a logically cohesive and integrative framework. We believe our results would pave the way for future studies to consider the collective effect of factors on the persistence phenomena and the relationships among the factors. An overview of the methodological framework used to conduct the review and the process adopted for categorizing factors in their respective categories is discussed in the later section.

## Methodological Framework

To understand the topic in-depth, we analyzed empirical studies published in peer-reviewed journals in the context of post-secondary education over the last two decades. Most of the review studies that focus on dropout/retention factors do not go beyond 10 years period. Ideally, the review on the subject should not miss any vital factor identified with the continuous evolution of the Internet, social, and educational technologies. This approach becomes significant when the intent is to arrange the factors into their logical categories and guide future studies to focus on the relationships among factors and their combined effect on persistence, while studying retention and dropout scenarios.

### Selection Criteria

Initially, the search phase explored Education Research Complete, ProQuest, ERIC, JSTOR, and PsycInfo databases, using the terms “online,” “persistence,” “dropout,” “retention,” “attrition,” and “withdrawal” in various combinations. Further, we searched with the same terms on Google Scholar and applied the snowball technique to enhance the existing pool. The screening phase concluded by analyzing the abstracts. Duplicates, non-empirical, non-peer-reviewed, and out-of-context studies were excluded.

### Method

**Figure 2:**
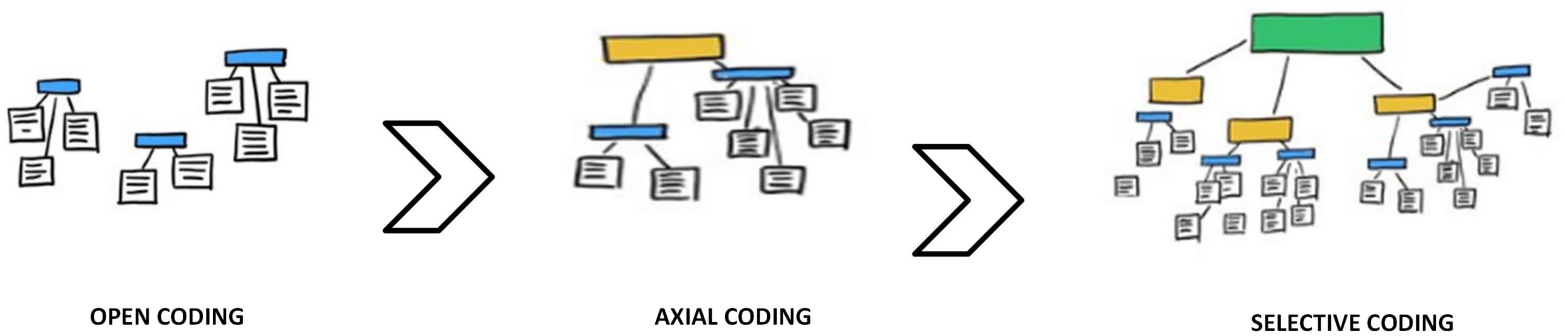
Pictorial representation of Constant Comparative Method (CCM).

After identifying the related factors from the final list of studies, we applied Constant Comparative Method (CCM) of Glaser and Strauss (1967, p. 102) to assign the factors into their logical categories. The constant comparative analysis is characterized by “explicit coding and analytic procedures.” Coding is the method of labeling and categorizing concepts. A concept can be viewed as a “basic unit of analysis” (Corbin and Strauss, 1990, p. 7). The formation of a category occurs when items with similar characteristics are grouped. There are three stages to coding: open, axial, and selective (Corbin and Strauss, 1990). In open coding, an incident is compared with other incidents based on their similarity and differences, Incidents are given conceptual labels, and the concepts are grouped into categories (Corbin and Strauss, 1990). Using axial coding, we explored the relationships between categories (Strauss, 1987). Authors have used selective coding to form a core category or categories and build a story that connects them. A pictorial representation of the process is given in Figure 2.

Our basic units of analysis (concepts) are the 47 individual factors identified through the literature review. Initially, we selected one factor randomly to represent the first category. Then, the similarity of the randomly chosen second factor with the previous factor was evaluated. If that second factor was not found to be similar to the first, we created a new category to represent the second factor. Two authors from this study judged the similarity of the factors to form categories of logically cohesive factors within them. We also consulted a peer de-briefer (subject expert) to mediate some of the differences between the authors in the process of factor assignment to their respective categories. The open coding process continued, creating 13 categories containing 47 individual factors. In the axial coding stage, the relationships are evaluated among the formed categories, forming the three axes (core categories), having 5, 5, and 3 categories in each axis, respectively (see Table 1).

**Table 1:** Summary of identified factors group wise.

Group name	Factors count	Categories count	Percent
Online learners	21	5	44%
Online courses and course providers	13	3	28%
Instructors	13	5	28%

## Review Results

The scope of this review comprises a reflection of factors that correlate positively or negatively to persistence in post-secondary online settings. Prior research on persistence and dropouts has not been comprehensive and integrative, utilizing divergent frameworks and approaches. Moreover, the categorization presented in previous studies has not considered the importance of the relationship between factors. The contribution of this paper is 2-fold. Firstly, we have identified all the factors linked to persistence reported for the past 20 years. Secondly, we have presented a logically coherent and integrative framework to enable fellow researchers to examine and understand the relationships among the persistence factors in future studies. There is a definite need to study the exact relationships among the persistence factors (Choi and Park, 2018). Therefore, we have focused on defining coherent categories of factors that can be used to analyze relationships among factors. Forming such categories can also provide essential insights for the institutes offering online courses, administrators of online programs, and course instructors/facilitators in improving retention and overall quality of online courses and programs.

### Persistence Factors Related to Online Learners

**Figure 3:**
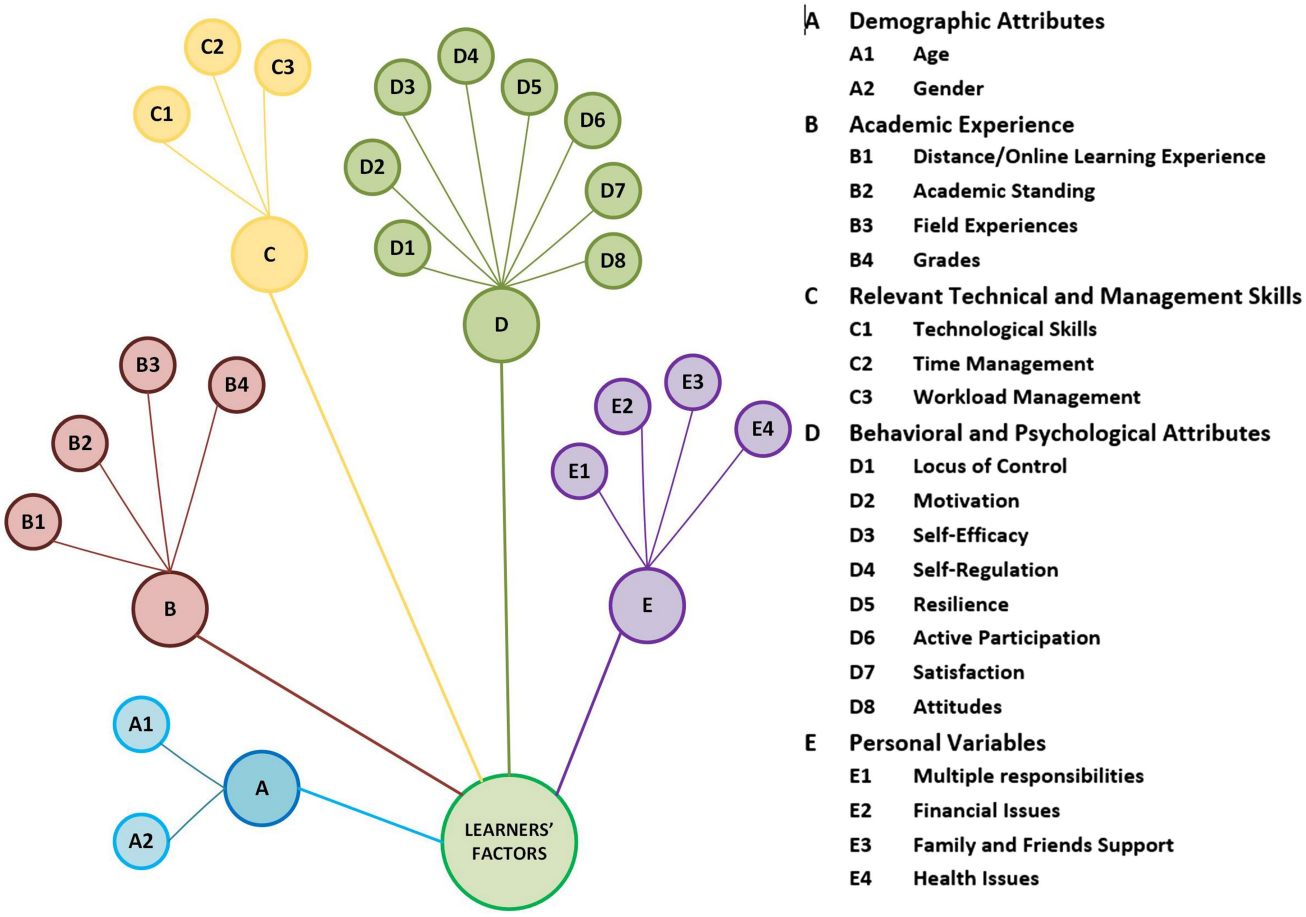
Categorical arrangement of factors related to online learners.

This section presents the factors related to online learners only. A review of these factors provides insights into the consensus among scholars, their differing views, and in some cases, contrasts empirical findings. The color-coded categorical arrangement of the factors related to online learners is presented in Figure 3.

#### Demographic Attributes

Most researchers have focused on the differences in age and gender concerning persistence or dropout decisions made by the learner.

##### Age

Some researchers reported no noteworthy difference in the age of students who drop out from online courses (Levy, 2007; Tello, 2008; Willging and Johnson, 2009; James et al., 2016), while others have noted age as an important factor (Xenos et al., 2002; Pierrakeas et al., 2004; Wladis et al., 2015; Murphy and Stewart, 2017). It has been posited that older students tend to drop out and require more encouragement from their teachers (Xenos et al., 2002). Conversely, a retention study for online (STEM) courses reveals that older students showed better performance and had more likelihood of persistence (Wladis et al., 2015). Similarly, James et al. (2016) stated that more senior students (age > 26) taking only online courses were retained more than younger students (age < 26). Also, Wuellner (2013) reported that younger learners might lack the skills and readiness required for online courses.

##### Gender

Some researchers believe that gender differences in online courses are not significantly related to retention/dropout (Parker, 1999; Kemp, 2002; Cochran et al., 2014; Wladis et al., 2015; James et al., 2016). However, some studies informed the likelihood of the male population dropping from online courses (Packham et al., 2004; Pocock et al., 2009). Studies also reveal that older female online learners get more influenced by the expectations around domestic and family responsibilities (Dupin-Bryant, 2004; Stone and O’Shea, 2013).

#### Academic Experience

Some aspects of academic experiences are linked with persistence and dropping out decisions by online learners.

##### Distance/Online Learning Experience

Previous experience with distance or online learning improves awareness and boosts confidence. The number of previously done online courses (Dupin-Bryant, 2004) and distant learning courses (Levy, 2007; Traver et al., 2014) has been found to be linked with persistence decisions.

##### Academic Standing

Academic standing in college (freshman, sophomore, junior, or senior) is found to be related to persistence in online classes. Learners with higher status have increased chances of persistence (Packham et al., 2004; Tello, 2008). However, Traver et al. (2014) has not found the academic year significant in predicting retention in online classes.

##### Field Experiences

While examining past educational and professional experiences of learners enrolled in an Informatics course online, Xenos et al. (2002) discovered that learners with prior backgrounds in programming or data handling showed significantly higher persistence rates. However, Cheung and Kan (2002) have not found previous experiences significant in persistence/dropout decisions.

##### Grades

Faculty and learners consider GPA and grades among the five most influential factors contributing to persistence/dropout decisions (Gaytan, 2015). Many researchers have indicated that learners with lower academic scores are most likely to drop out of online classes (Packham et al., 2004; Aragon and Johnson, 2008; Harrell and Bower, 2011; Xu and Jaggars, 2011; Colorado and Eberle, 2012; Stewart et al., 2013). Conversely, others have not found grades very significant in predicting retention/dropout (Hachey et al., 2013; Traver et al., 2014; Shaw et al., 2016).

#### Relevant Technical and Management Skills

Previous research has focused on various technical and management skills of online learners that are found to be linked with persistence in online courses.

##### Technological Skills

Technological skills and confidence in using the computer, college readiness, and clarity of goals influence completing an online course (Traver et al., 2014; Blau et al., 2016). The absence or lack of technical skills related to the Internet and its applications, operating systems, and file management is an important dropout indicator (Dupin-Bryant, 2004). Similarly, Blau et al. (2016) found perceived ease of using technology is linked with persistence.

##### Time Management

While effective time management skills have been reported to influence persistence positively, learners’ difficulty in managing time has been strongly associated with early dropouts from online classes (Ivankova and Stick, 2007; Stanford-Bowers, 2008; Nichols, 2010; Traver et al., 2014). Good study habits such as prioritizing tasks like assignments and making efficient use of available time enable learners to continue (Castles, 2004; Ivankova and Stick, 2007). Aragon and Johnson (2008) supported this finding but noted a modest difference in the students’ capability enrolled in more online courses. The skill and ability to balance multiple responsibilities have been seen in those learners who complete their online courses (Müller, 2008; Joo et al., 2011). Realistic expectations about the time and effort to complete a task are reported to facilitate better academic performance and completion of online courses (Xenos et al., 2002; Wladis et al., 2015).

##### Workload Management

Online learners who actively plan to accommodate their workload are more likely to persist (Bunn, 2004). Realistic expectations about the workload are noted as facilitators of persistence (Leeds et al., 2013). An unexpected change in the workload of an online class is also reported as a dropout reason (Moore and Greenland, 2017).

#### Behavioral and Psychological Attributes

Online learners’ behavioral and psychological characteristics encompass various attitudes and traits that shape their decision to persist or drop out.

##### Locus of Control

Thoughts about where to attribute outcomes of an event and the level of control over that subsequent event (Rotter, 1966) is an individual’s locus of control. Lee and Choi (2013) found the locus of control as an influencing factor related to persistence. Individuals who have an “internal locus of control” tend to believe that the result of actions depends on their decisions and effort. Internal locus of control has been reported to link with persistence in online courses (Parker, 2003; Morris et al., 2005b).

##### Motivation

It is the most significant force that shapes learners’ perceptions about enrolling in online classes and helps them persist (Kemp, 2002; Holder, 2007; Blau et al., 2016). Motivation can positively forecast dropout decisions (Osborn, 2001). Self-motivation, alongside personal challenge and responsibility, is considered the intrinsic motivation to conclude an online program (Park and Choi, 2009; Nichols, 2010). Attachment and commitment toward a goal, goal attainment, respect for career, and financial outcomes of education are linked with persistence in online education (Nichols, 2010; Joo et al., 2011). Self-determination helps to sustain learners in the online program (Nichols, 2010).

##### Self-Efficacy

It is a “belief that one is capable of executing certain behaviors or achieving certain goals” (Ormrod, 2011, p. 352). Online student self-efficacy is identified as the most influential factor linked to retention (Ivankova and Stick, 2007; Liaw, 2008; Street, 2010). A higher level of self-efficacy increases resilience in the cases of obstacles and intensifies learners’ efforts (Kemp, 2002). Learners’ endurance to complete is associated with self-regulation and self-efficacy (Gomez, 2013). Similarly, Ivankova and Stick (2007) and Ice et al. (2011) indicated a significant correlation between online course completion and self-efficacy.

##### Self-Regulation

It is an individual ability to control behavior, emotions, and thoughts in the engagement toward long-term goals. Those online learners who “self-regulate” successfully practice metacognitive, motivational, and behavioral processes as part of forethought, performance, and self-reflection (Zimmerman, 2011). These behaviors generally include effective time management, seeking help from online course facilitators or tutoring, and avoiding distractions. Self-regulation influences learners’ persistence (Gomez, 2013; Lee et al., 2013; O’Neill and Sai, 2014). Similarly, Lee et al. (2013) report meta-cognition as an influencing factor linked with retention. Self-discipline is also an influential factor contributing to persistence (Gaytan, 2015).

##### Resilience

An ability to manage threats during online courses has been an influencing factor differentiating persistent students from dropouts (Parker, 1999; Müller, 2008).

##### Active Participation

Although a mild relationship exists between learner participation and academic success in terms of final grades (Xia et al., 2013), online learners who actively interact with the course content are more likely to persist. Learners who complete their course view more discussion/content pages and spend more time viewing the discussions than those who withdraw (Morris et al., 2005a).

##### Satisfaction

Satisfaction with faculty and online courses has been found to be correlated with course completion in previous studies (Tello, 2008; Joo et al., 2011).

##### Attitudes

Learners’ attitudes toward the course and their interactions with fellow peers and facilitators (instructors) are correlated with the completion of online courses (Tello, 2008).

#### Personal Variables

##### Multiple Responsibilities

Family responsibilities are seen as a hindrance and a reason to withdraw from online learning in past studies (Parkes et al., 2015; Shah and Cheng, 2019). Employment responsibilities also create problems for learners to continue (Lee and Choi, 2011; Shah and Cheng, 2019), and part-time learners tend to drop out more from online classes (Boston et al., 2011).

##### Financial Issues

Issues related to finance may contribute to dropout decisions by online learners (Aversa and MacCall, 2013; Parkes et al., 2015). Online students usually pay the tuition fees out of pocket, and this added responsibility influences persistence decisions (Boston et al., 2011). Contradictorily, Cochran et al. (2014) state that learners with loans/financial assistance are more inclined to drop out having certain major subjects.

##### Family and Friends Support

Family support and home environment is also significant factor related to persistence (Harris et al., 2011). Non-persistent learners see friends and family as unsupportive in their educational journey (Park and Choi, 2009). Learners who persist score higher in having supportive partners and maintaining healthy relationships (Kemp, 2002).

##### Health Issues

Issues related to disability and health may also cause online learners to withdraw (Shah and Cheng, 2019).

### Persistence Factors Related to Online Courses and Course Providers

**Figure 4:**
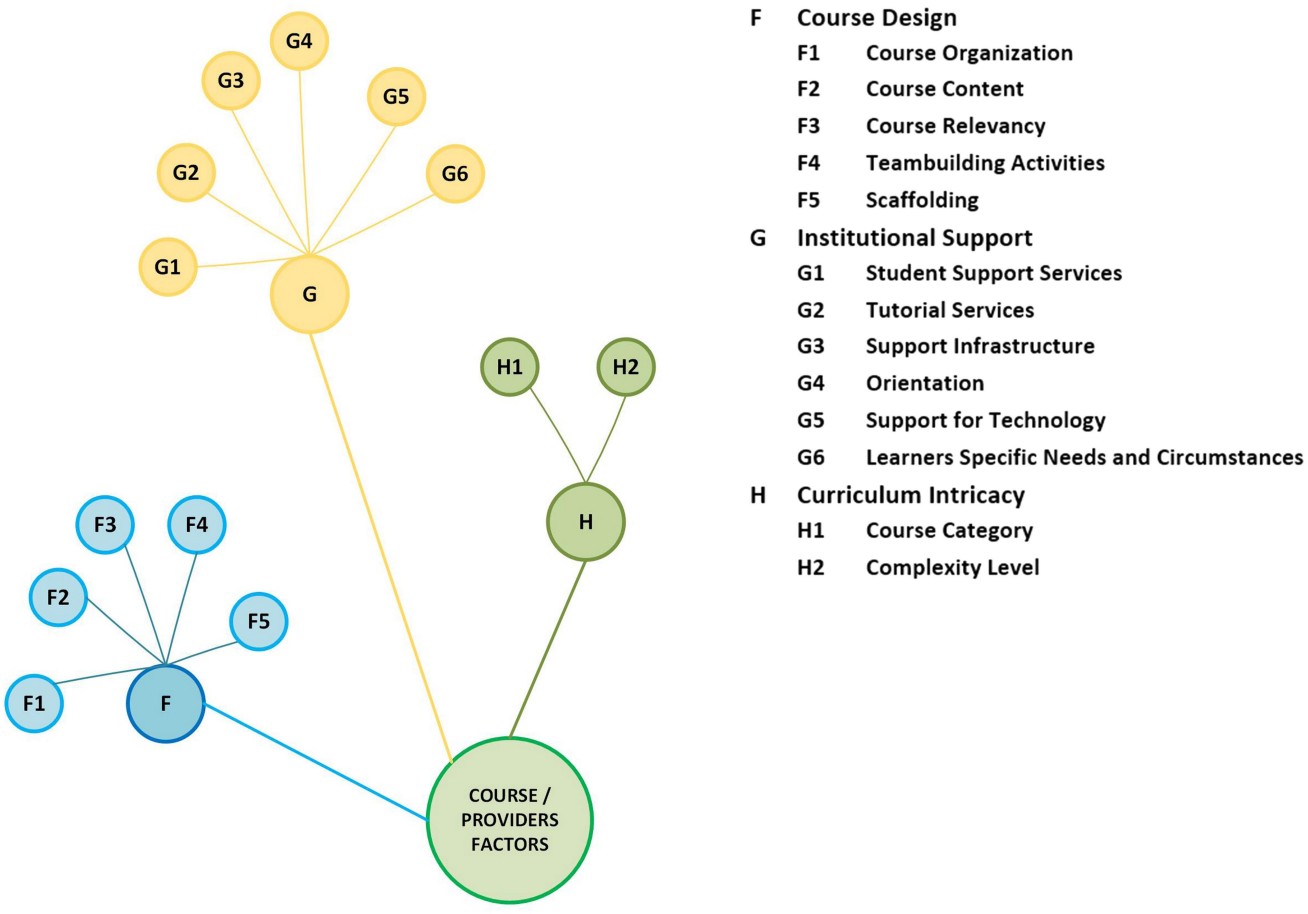
Categorical arrangement of factors related to online courses and course providers.

Factors linked with online course design and institutional support are listed in this section. This includes how the course or program is structured, the complexity of the curriculum, how the learners interact with the content, and what support services they perceive important. The color-coded categorical arrangement of the factors related to online courses and course providers is presented in Figure 4.

#### Course Design

How the courses are defined and structured in terms of their interactivity, how well they fulfill the need of the learners, and the overall quality of online courses are important predictors of persistence and dropout.

##### Course Organization

Bad course organization, or at worst, lack of course organization and disconnected, illogical structures of the courses are linked with dropout decisions (Hammond and Shoemaker, 2014). Ice et al. (2011) noted that poor course design/organization affects learner satisfaction, thus contributing to dropout decisions.

##### Course Content

Well-structured courses with rigorous, relevant content and clear instructions facilitate persistence (Nichols, 2010; Harris et al., 2011), whereas boring and unrelated course elements promote dropout decisions (Pittenger and Doering, 2010; Garratt-Reed et al., 2016).

##### Course Relevancy

Course relevancy with individuals’ learning styles and career objectives is important in shaping their decision to persist or withdraw from online courses (Perry et al., 2008). Street (2010) also points out that relevant course factors and design impact learners’ choice to continue or drop out.

##### Team-Building Activities

Courses that promote team-building activities foster increased interaction between the learners and the faculty, thus contributing to increased retention (Bocchi et al., 2004).

##### Scaffolding

An element of scaffolding fused into the course design forms striking, motivating, and related learning elements that enhance persistence (Pittenger and Doering, 2010).

#### Institutional Support

Institutional support services have been confirmed crucial for online course completion by the administrators and faculty (Heyman, 2010; Boston et al., 2011). However, learners do not perceive these support services as equally important (Gaytan, 2015) but admit that the absence of these services negatively impacts their academic success (Nichols, 2010).

##### Student Support Services

These services help learners overcome barriers that result in dropout decisions. Xu and Jaggers (2011) confirms that support services for online learners are not found as effective or satisfactory as they are for regular students. However, Muilenburg and Berge (2001) acknowledged unsatisfactory support services as barriers for online learners.

##### Tutorial Services

The academic and emotional support provided to online learners through face-to-face sessions improved persistence in online courses significantly (Levy, 2007). Similarly, online learners perceive tutorials as helpful, encouraging them to continue (Stanford-Bowers, 2008).

##### Support Infrastructure

Muilenburg and Berge (2001) conducted a factor analysis to study barriers related to distance education and identified a 10-factor model that deters course completion. Among these, five factors were found linked to institutional support infrastructure. These five factors are: (1) Structure of administration; (2) Student-support services; (3) Access; (4) Effectiveness and Evaluation; and (5) Teacher compensation and time. These factors were confirmed to influence distant learners’ dropping out decisions through telephonic interviews (Clay et al., 2008; Nichols, 2010).

##### Orientation

Course orientation facilitates the chances of online learners persisting in the course (Clay et al., 2008; Aversa and MacCall, 2013). Online advisory counseling and web orientation provided to undergraduates significantly increase the persistence rate (Clay et al., 2008).

##### Support for Technology

Online learners possess different levels of skills related to computers and technology, and the perception of being unsupported is more of a problem than the actual struggle with technology (Bunn, 2004). Parkes et al. (2015) exposed insufficient technology support to distant learners, impacting persistence (Ojokheta, 2010; Street, 2010). However, Ivankova and Stick (2007) have not found technical support influential but agree that non-persistent learners were not pleased with the support services. Also, it is revealed that access issues with technology and the poor speed of the Internet may also influence dropout decisions (Osborn, 2001).

##### Learners’ Specific Needs and Circumstances

Institutional lack of understanding of online learners’ needs and their specific circumstances contribute to dropout decisions (Parkes et al., 2015; Friðriksdóttir, 2018).

#### Curriculum Intricacy

The category of an online course and its complexity level has been noted as influencing elements linked to learners’ persistence.

##### Course Category

The category of the course (elective, distribution, and major) and retention in online settings are interlinked (Wladis et al., 2017). Additionally, Wladis et al. (2014) found lower-level STEM courses and dropout rates were positively associated.

##### Complexity Level

Online learners tend to drop out of online programs if there are many low-level and easy assignments or if they find the program curriculum too difficult (Willging and Johnson, 2009). Similarly, Boston et al. (2011) posit that online learners were more inclined to drop out if they find the curriculum very easy or very difficult.

### Persistence Factors Related to Online Instructors

**Figure 5:**
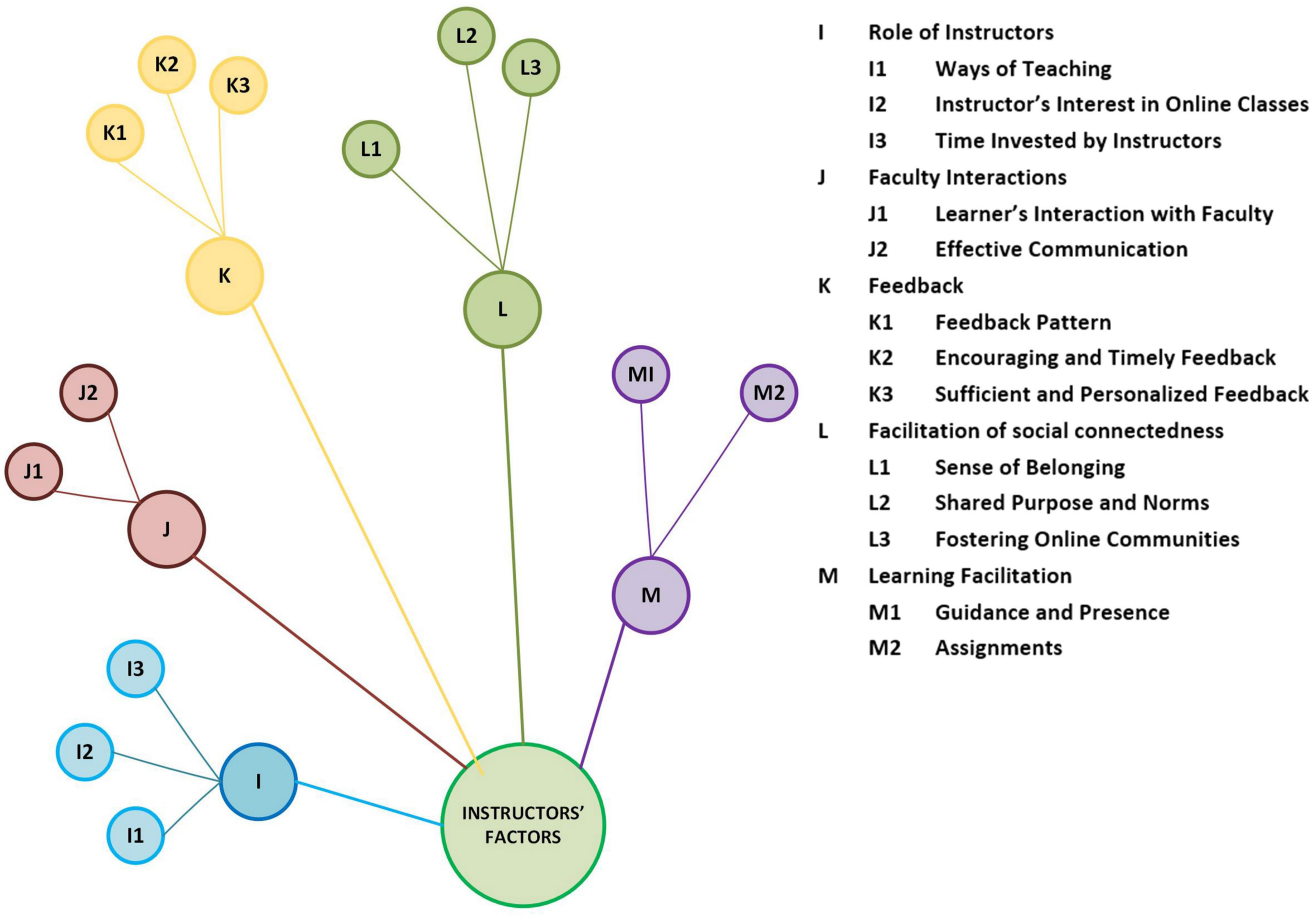
Categorical arrangement of factors related to online course instructors.

Universities need to inspire faculty to develop themselves to improve the quality of online courses (Parker et al., 2013). The role of online course facilitators is vital in keeping learners’ interests intact, keeping them motivated to continue, and helping them to conclude online courses and programs successfully. The color-coded categorical arrangement of the factors related to online course instructors is presented in Figure 5.

#### Role of Instructors

The instructor’s role is evolving in online settings, and this change is observed as a significant challenge (Syverson and Slatin, 2010).

##### Ways of Teaching

The main challenge of teaching online courses is the “disconnect between the way teachers were taught to teach” (Anderson et al., 2011, p. 4). The shift toward the learner-centered approach has transformed the role of instructors into guides with the responsibility to align the content delivery according to the need of the learners.

##### Instructor’s Interest in Online Classes

Instructors involved in traditional face-to-face classes are found uninterested in teaching in online settings, fearing that they are replaceable with computers (Osika et al., 2009). Müller (2008) has identified that students’ dissatisfaction with faculty or learning results in dropouts. More time required in preparation, design, and facilitation may also limit the interest of the faculty in online classes (Crawley et al., 2009).

##### Time Invested by Instructors

Preparing for, planning, and teaching an online class took an extra bit of time (Capra, 2011), and the amount of time spent by the instructors, while facilitating online courses are linked with student retention up to a certain extent (Wuellner, 2013).

#### Faculty Interactions

Interaction with faculty has been nominated as the second-highest retention factor, the absence of which contributes to dissatisfaction and dropout decisions in online learning (Heyman, 2010; Boston et al., 2011).

##### Learner’s Interaction With Faculty

Interaction of online learners with the faculty and dropout rates are significantly linked (Bocchi et al., 2004).

##### Effective Communication

Online learners expect effective communication from the course facilitators, and its absence creates difficulties for them to persist (O’Neill and Sai, 2014). Online learners who interact effectively with the faculty persist more (Ivankova and Stick, 2007).

#### Feedback

Feedback from the faculty, association, motivation, and perception is positively associated with online learners’ outcomes (Ojokheta, 2010).

##### Feedback Pattern

Feedback from faculty influence the perception of students regarding course content, and feedback pattern directly affects their ability to conclude an online course positively (Ojokheta, 2010).

##### Encouraging and Timely Feedback

Positive, timely, valuable, encouraging feedback and faculty readiness to meet learner needs are significant to students’ persistence (Ivankova and Stick, 2007).

##### Sufficient and Personalized Feedback

Insufficient or inadequate feedback on learning affects retention (Shah and Cheng, 2019). Feedback should be consistent and personalized for each student (Bocchi et al., 2004).

#### Facilitation of Social Connectedness

A sense of social connectedness fosters interaction with peers and the learning community. It is possible for online learners to feel disconnected and isolated (McInnerney and Roberts, 2004), negatively affecting their overall learning experience and persistence.

##### Sense of Belonging

Apparently, verbal and visual communication cues are not displayed in online learning environments as in traditional settings (Koole, 2014), resulting in isolation and not being supported by peers (Aversa and MacCall, 2013; Koole, 2014). This negative perception is linked with an inferior sense of community and deprived student bonding (Aversa and MacCall, 2013) that create difficulties in breaking the ice between peers, thus influencing their decision to persist. Associating themselves with the learning community instigates learners’ sense of identity and inspires their learning (Koole, 2014).

##### Shared Purpose and Norms

Online learners should be assisted in developing shared purpose and norms and a fit-in sense (Lapadat, 2007; Nistor and Neubauer, 2010). Learners who do not share a common purpose and community norms usually fail to interact actively, stay quiet during discussions, and are more persuaded to drop out (Nistor and Neubauer, 2010).

##### Fostering Online Communities

An essential role of online instructors is to promote and encourage an online community (Drouin, 2008; Nichols, 2010), assure peer interactions (Pigliapoco and Bogliolo, 2008; Alman et al., 2012), and facilitate effective dialogs with peers (Alman et al., 2012). Becoming a valuable part of the knowledge community fosters an effective knowledge construction process, thus increasing learners’ chances of persistence (Goodyear and Zenios, 2007).

#### Learning Facilitation

One key role of online instructors is to assist online learners in generating and achieving knowledge, facilitating the overall learning process.

##### Guidance and Presence

Online learners value instructors’ presence in nurturing the knowledge attainment process (Alman et al., 2012). Insufficient advice about the topics is linked with low online enrolment (Ice et al., 2011).

##### Assignments

The type of assignment presented to online learners could also affect learners’ decisions to continue with the course. Fredrickson (2015) and Garratt-Reed et al. (2016) highlights that online learners do not prefer group assignments because of limited personal interaction with the course instructor.

## Conclusion

This review reflected upon the essential factors linked with persistence, either positively or negatively, by methodically reviewing empirical studies on the subject published in the past two decades. By applying the CCM method, we managed to classify the identified factors into three broad groups, each one containing sub-groups of factors within them. Factors related to online learners are presented in the first group having demographic properties, past educational experiences, management and technological skills, behavioral and psychological attributes of the learner, and other personal variables related to responsibilities, support, health, and finances. Persistence factors related to online learners are most discussed in the reviewed studies. Factors related to online course design and structure, support from the online course providers, and the complexity level of online courses and programs are placed in the second group. Finally, the third group presents factors related to online course instructors like their role in online settings, how well they facilitate online learning, their role in promoting various interactions, and their interest in the online mode of delivery.

Researchers found that the interaction between various factors determines whether online learners persist or drop out (Holder, 2007; Perry et al., 2008). Therefore, the categorization provided in this review will help fellow researchers to investigate the relationship within and between categories alongside studying the combined effect of various factors on persistence or dropout decisions. The results will direct future research to critically examine the relationships among the factors and suggest improvements by validating them empirically. Future researchers may also validate the results in different scenarios and contexts related to online learning. Course instructors and providers can focus on the related problem areas to improve online courses and programs persistence.

## Author Contributions

US: conception, design of the work, data collection, drafting the article, and critical revision of the article. ZA: conceptualization, design review, draft review, and improvement suggestions. US and ZA: data analysis and interpretation and final approval of the version to be published. All authors contributed to the article and approved the submitted version.

## Conflict of Interest

The authors declare that the research was conducted in the absence of any commercial or financial relationships that could be construed as a potential conflict of interest.

## Publisher’s Note

All claims expressed in this article are solely those of the authors and do not necessarily represent those of their affiliated organizations, or those of the publisher, the editors and the reviewers. Any product that may be evaluated in this article, or claim that may be made by its manufacturer, is not guaranteed or endorsed by the publisher.

## References

[ref1] Alman S. W. Frey B. A. Tomer C. (2012). Social and cognitive presence as factors in learning and student retention: an investigation of the cohort model in an iSchool setting. J. Educ. Libr. Inf. Sci. 290–302.

[ref2] Almanthari A. Maulina S. Bruce S. (2020). Secondary school mathematics teachers’ views on e-learning implementation barriers during the COVID-19 pandemic: the case of Indonesia. Eurasia J. Math. Sci. Technol. Educ. 16:em1860. doi: 10.29333/ejmste/8240

[ref3] Anderson D. Imdieke S. Standerford N. S. (2011). Feedback please: studying self in the online classroom. Int. J. Instr. 4, 3–15.

[ref4] Aragon S. R. Johnson E. S. (2008). em0060Factors influencing completion and noncompletion of community college online courses. Am. J. Dist. Educ. 22, 146–158. doi: 10.1080/08923640802239962

[ref5] Aversa E. MacCall S. (2013). Profiles in retention part 1: design characteristics of a graduate synchronous online program. J. Educ. Libr. Inf. Sci. 147–161.

[ref6] Basilaia G. Kvavadze D. (2020). Transition to online education in schools during a SARS-CoV-2 coronavirus (COVID-19) pandemic in Georgia. Pedagogical Res. 5:em0060. doi: 10.29333/pr/7937

[ref7] Blau G. Drennan R. B. Hochner A. Kapanjie D. (2016). Perceived learning and timely graduation for business undergraduates taking an online or hybrid course. J. Educ. Bus. 91, 347–353. doi: 10.1080/08832323.2016.1218319, PMID: 35436160

[ref8] Bocchi J. Eastman J. K. Swift C. O. (2004). Retaining the online learner: profile of students in an online MBA program and implications for teaching them. J. Educ. Bus. 79, 245–253. doi: 10.3200/JOEB.79.4.245-253

[ref9] Boston W. E. Ice P. Gibson A. M. (2011). Comprehensive assessment of student retention in online learning environments. Online J. Dist. Learn. Admin. 14.

[ref10] Bowden J. (2008). Why do nursing students who consider leaving stay on their courses? Nurs. Res. 15, 45–58. doi: 10.7748/nr2008.04.15.3.45.c6456, PMID: 18459486

[ref11] Bunn J. (2004). Student persistence in a LIS distance education program. Austral. Acad. Res. Librar. 35, 253–269. doi: 10.1080/00048623.2004.10755275

[ref12] Capra T. (2011). Online education: promise and problems. J. Online Learn. Teach. 7, 288–293.

[ref13] Castles J. (2004). Persistence and the adult learner: factors affecting persistence in open university students. Act. Learn. High. Educ. 5, 166–179. doi: 10.1177/1469787404043813

[ref01] Cheung L. L. Kan A. C. (2002). Evaluation of factors related to student performance in a distance-learning business communication course. J. Educ. Bus. 77, 471 257–263. doi: 10.1080/08832320209599674

[ref14] Chiyaka E. T. Sithole A. Manyanga F. McCarthy P. Bucklein B. K. (2016). Institutional characteristics and student retention: what integrated postsecondary education data reveals about online learning. Online J. Dist. Learn. Admin. 19.

[ref15] Choi H. J. Park J.-H. (2018). Testing a path-analytic model of adult dropout in online degree programs. Comput. Educ. 116, 130–138. doi: 10.1016/j.compedu.2017.09.005

[ref16] Clay M. N. Rowland S. Packard A. (2008). Improving undergraduate online retention through gated advisement and redundant communication. J. Coll. Stud. Retent. Res. Theory Pract. 10, 93–102. doi: 10.2190/CS.10.1.g

[ref17] Cochran J. D. Campbell S. M. Baker H. M. Leeds E. M. (2014). The role of student characteristics in predicting retention in online courses. Res. High. Educ. 55, 27–48. doi: 10.1007/s11162-013-9305-8

[ref02] Colorado J. T. Eberle J. (2012). Student demographics and success in online learning 486 environments. https://dspacep01.emporia.edu/bitstream/handle/123456789/380/205.2.pdf

[ref18] Corbin J. M. Strauss A. (1990). Grounded theory research: procedures, canons, and evaluative criteria. Qual. Sociol. 13, 3–21. doi: 10.1007/BF00988593

[ref19] Crawley F. E. Fewell M. D. Sugar W. A. (2009). Researcher and researched: the phenomenology of change from face-to-face to online instruction. Quart. Rev. Dist. Educ. 10, 165–176.

[ref20] Delnoij L. E. Dirkx K. J. Janssen J. P. Martens R. L. (2020). Predicting and resolving non-completion in higher (online) education–a literature review. Educ. Res. Rev. 29:100313. doi: 10.1016/j.edurev.2020.100313

[ref21] Drouin M. A. (2008). The relationship between students’perceived sense of community and satisfaction, achievement, and retention in an online course. Quart. Rev. Dist. Educ. 9

[ref22] Dupin-Bryant P. A. (2004). Pre-entry variables related to retention in online distance education. Am. J. Dist. Educ. 18, 199–206. doi: 10.1207/s15389286ajde1804_2

[ref23] Fredrickson J. (2015). Online learning and student engagement: assessing the impact of a collaborative writing requirement. Acad. Educ. Leadersh. J. 19, 127–140.

[ref24] Friðriksdóttir K. (2018). The impact of different modalities on student retention and overall engagement patterns in open online courses. Comput. Assist. Lang. Learn. 31, 53–71. doi: 10.1080/09588221.2017.1381129

[ref25] Garratt-Reed D. Roberts L. D. Heritage B. (2016). Grades, student satisfaction and retention in online and face-to-face introductory psychology units: a test of equivalency theory. Front. Psychol. 7:673. doi: 10.3389/fpsyg.2016.00673, PMID: 27242587 PMC4862241

[ref26] Gaytan J. (2013). Factors affecting student retention in online courses: overcoming this critical problem. Career Tech. Educ. Res. 38, 145–155. doi: 10.5328/cter38.2.147

[ref27] Gaytan J. (2015). Comparing faculty and student perceptions regarding factors that affect student retention in online education. Am. J. Dist. Educ. 29, 56–66. doi: 10.1080/08923647.2015.994365

[ref28] Gazza E. A. Hunker D. F. (2014). Facilitating student retention in online graduate nursing education programs: a review of the literature. Nurse Educ. Today 34, 1125–1129. doi: 10.1016/j.nedt.2014.01.010, PMID: 24529796

[ref29] Glaser B. Strauss A. L. (1967). The Discovery of Grounded Theory. Chicago: Aldine.

[ref30] Gomez D. (2013). Leadership behavior and its impact on student success and retention in online graduate education. Acad. Educ. Leadersh. J. 17, 13–37.

[ref31] Goodyear P. Zenios M. (2007). Discussion, collaborative knowledge work and epistemic fluency. Br. J. Educ. Stud. 55, 351–368. doi: 10.1111/j.1467-8527.2007.00383.x

[ref03] Hachey A. C. Wladis C. W. Conway K. M. (2013). Balancing retention and access in online 531 courses: Restricting enrollment. Is it worth the cost?. J. Coll. Stud. Retent. Res. Theory Pract. 15, 9–36. doi: 10.2190/CS.15.1.b

[ref32] Hammond D. E. Shoemaker C. (2014). Are there differences in academic and social integration of college of agriculture master’s students in campus based, online and mixed programs? NACTA J. 58, 180–188.

[ref33] Harris S. M. Larrier Y. I. Castano-Bishop M. (2011). Development of the student expectations of online learning survey (SEOLS): a pilot study. Online J. Dist. Learn. Admin. 14:6

[ref04] Harrell I. L. Bower B. L. (2011). Student characteristics that predict persistence in community college online courses. American Journal of Distance Education 25, 178–191. doi: 10.1080/08923647.2011.590107

[ref34] Heyman E. (2010). Overcoming student retention issues in higher education online programs. Online J. Dist. Learn. Admin. 13:11.

[ref35] Hobson T. D. Puruhito K. K. (2018). Going the distance: online course performance and motivation of distance learning students. Online Learn. 22, 129–140. doi: 10.24059/olj.v22i4.1516

[ref36] Holder B. (2007). An investigation of hope, academics, environment, and motivation as predictors of persistence in higher education online programs. Internet High. Educ. 10, 245–260. doi: 10.1016/j.iheduc.2007.08.002

[ref37] Ice P. Gibson A. M. Boston W. Becher D. (2011). An exploration of differences between community of inquiry indicators in low and high disenrollment online courses. J. Asynchron. Learn. Netw. 15, 44–69. doi: 10.24059/olj.v15i2.196

[ref38] Ivankova N. V. Stick S. L. (2007). Students’ persistence in a distributed doctoral program in educational leadership in higher education: a mixed methods study. Res. High. Educ. 48, 93–135. doi: 10.1007/s11162-006-9025-4

[ref39] James S. Swan K. Daston C. (2016). Retention, progression and the taking of online courses. Online Learn. 20, 75–96. doi: 10.24059/olj.v20i2.780

[ref40] Johnson N. Bates T. Donovan T. Seaman J. (2019). Tracking Online Education in Canadian Universities and Colleges: National Survey of Online and Digital Learning 2019 National Report.

[ref41] Joo Y. J. Lim K. Y. Kim E. K. (2011). Online university students’ satisfaction and persistence: examining perceived level of presence, usefulness and ease of use as predictors in a structural model. Comput. Educ. 57, 1654–1664. doi: 10.1016/j.compedu.2011.02.008

[ref42] Kaur G. (2020). Digital life: boon or bane in teaching sector on COVID-19. CLIO Annu. Interdiscip. J. History 6, 416–427.

[ref43] Kemp W. C. (2002). Persistence of adult learners in distance education. Am. J. Dist. Educ. 16, 65–81. doi: 10.1207/S15389286AJDE1602_2

[ref44] Koole M. (2014). Identity and the itinerant online learner. Int. Rev. Res. Open Dist. Learn. 15, 52–70. doi: 10.19173/irrodl.v15i6.1879

[ref45] Kumar P. Kumar A. Palvia S. Verma S. (2019). Online business education research: systematic analysis and a conceptual model. Int. J. Manag. Educ. 17, 26–35. doi: 10.1016/j.ijme.2018.11.002

[ref46] Lapadat J. (2007). Discourse devices used to establish community, increase coherence, and negotiate agreement in an online university course. J. Dist. Educ. 21, 59–92.

[ref47] Lee K. (2017). Rethinking the accessibility of online higher education: a historical review. Internet High. Educ. 33, 15–23. doi: 10.1016/j.iheduc.2017.01.001

[ref48] Lee Y. Choi J. (2011). A review of online course dropout research: implications for practice and future research. Educ. Technol. Res. Dev. 59, 593–618. doi: 10.1007/s11423-010-9177-y

[ref49] Lee Y. Choi J. (2013). A structural equation model of predictors of online learning retention. Internet High. Educ. 16, 36–42. doi: 10.1016/j.iheduc.2012.01.005

[ref50] Lee Y. Choi J. Kim T. (2013). Discriminating factors between completers of and dropouts from online learning courses. Br. J. Educ. Technol. 44, 328–337. doi: 10.1111/j.1467-8535.2012.01306.x

[ref51] Leeds E. Campbell S. Baker H. Ali R. Brawley D. Crisp J. (2013). The impact of student retention strategies: an empirical study. Int. J. Manag. Educ. 7, 22–43. doi: 10.1504/IJMIE.2013.050812, PMID: 26828834

[ref52] Levy Y. (2007). Comparing dropouts and persistence in e-learning courses. Comput. Educ. 48, 185–204. doi: 10.1016/j.compedu.2004.12.004

[ref53] Li K. C. Wong B. T.-M. (2019). Factors related to student persistence in open universities: changes over the years. Int. Rev. Res. Open Dist. Learn. 20, 132–151. doi: 10.19173/irrodl.v20i4.4103

[ref54] Liaw S.-S. (2008). Investigating students’ perceived satisfaction, behavioral intention, and effectiveness of e-learning: a case study of the blackboard system. Comput. Educ. 51, 864–873. doi: 10.1016/j.compedu.2007.09.005

[ref55] Limperos A. M. Buckner M. M. Kaufmann R. Frisby B. N. (2015). Online teaching and technological affordances: an experimental investigation into the impact of modality and clarity on perceived and actual learning. Comput. Educ. 83, 1–9. doi: 10.1016/j.compedu.2014.12.015

[ref56] Maqsood A. Abbas J. Rehman G. Mubeen R. (2021). The paradigm shift for educational system continuance in the advent of COVID-19 pandemic: mental health challenges and reflections. Curr. Res. Behav. Sci. 2:100011. doi: 10.1016/j.crbeha.2020.100011 PMC7832654 38620741

[ref57] McInnerney J. M. Roberts T. S. (2004). Online learning: social interaction and the creation of a sense of community. J. Educ. Technol. Soc. 7, 73–81.

[ref58] Mishra S. (2017). Open Universities in the Commonwealth: At a Glance. Availble at: http://hdl.handle.net/11599/2786 (Accessed August 21, 2021).

[ref59] Moore C. Greenland S. (2017). Employment-driven online student attrition and the assessment policy divide: an Australian open-access higher education perspective. J. Open Flexible Dist. Learn. 21:52

[ref60] Morris L. V. Finnegan C. Wu S.-S. (2005a). Tracking student behavior, persistence, and achievement in online courses. Internet High. Educ. 8, 221–231. doi: 10.1016/j.iheduc.2005.06.009

[ref61] Morris L. V. Wu S.-S. Finnegan C. L. (2005b). Predicting retention in online general education courses. Am. J. Dist. Educ. 19, 23–36. doi: 10.1207/s15389286ajde1901_3

[ref62] Muilenburg L. Berge Z. L. (2001). Barriers to distance education: a factor-analytic study. Am. J. Dist. Educ. 15, 7–22. doi: 10.1080/08923640109527081

[ref63] Muljana P. S. Luo T. (2019). Factors contributing to student retention in online learning and recommended strategies for improvement: a systematic literature review. J. Inform. Technol. Educ. Res. 18, 19–57. doi: 10.28945/4182

[ref64] Müller T. (2008). Persistence of women in online degree-completion programs. Int. Rev. Res. Open Dist. Learn. 9, 1–18. doi: 10.19173/irrodl.v9i2.455

[ref65] Murphy C. A. Stewart J. C. (2017). On-campus students taking online courses: factors associated with unsuccessful course completion. Internet High. Educ. 34, 1–9. doi: 10.1016/j.iheduc.2017.03.001

[ref66] Nichols M. (2010). Student perceptions of support services and the influence of targeted interventions on retention in distance education. Distance Educ. 31, 93–113. doi: 10.1080/01587911003725048

[ref67] Nistor N. Neubauer K. (2010). From participation to dropout: quantitative participation patterns in online university courses. Comput. Educ. 55, 663–672. doi: 10.1016/j.compedu.2010.02.026

[ref68] O’Neill D. K. Sai T. H. (2014). Why not? Examining college students’ reasons for avoiding an online course. High. Educ. 68, 1–14. doi: 10.1007/s10734-013-9663-3

[ref69] Ojokheta K. O. (2010). A path-analytic study of some correlates predicting persistence and student’s success in distance education in Nigeria. Turk. Online J. Dist. Educ. 11, 181–192.

[ref70] Ormrod J. E. (2011). “Social cognitive views of learning,” in Educational Psychology: Developing Learners. ed. Smith P. A., 352–354.

[ref71] Osborn V. (2001). Identifying at-risk students in videoconferencing and web-based distance education. Am. J. Dist. Educ. 15, 41–54. doi: 10.1080/08923640109527073

[ref72] Osika E. Johnson R. Butea R. (2009). Factors influencing faculty use of technology in online instruction: a case study. Online J. Dist. Learn. Admin. 12, 1–14.

[ref73] Packham G. Jones P. Miller C. Thomas B. (2004). E-learning and retention: key factors influencing student withdrawal. Educ. Train. 46, 335–342. doi: 10.1108/00400910410555240

[ref74] Park J.-H. Choi H. J. (2009). Factors influencing adult learners’ decision to drop out or persist in online learning. J. Educ. Technol. Soc. 12, 207–217.

[ref75] Parker A. (1999). A study of variables that predict dropout from distance education. Int. J. Educ. Technol. 1, 1–10.

[ref76] Parker A. (2003). Identifying predictors of academic persistence in distance education. USDLA J. 17, 55–62.

[ref77] Parker J. Maor D. Herrington J. (2013). “Authentic online learning: aligning learner needs, pedagogy and technology,” in Issues in Educational Research, *Vol*. 23, 227.

[ref78] Parkes M. Gregory S. Fletcher P. Adlington R. Gromik N. (2015). Bringing people together while learning apart: creating online learning environments to support the needs of rural and remote students. Aust. Int. J. Rural Educ. 25, 65–78.

[ref79] Perry B. Boman J. Care W. D. Edwards M. Park C. (2008). Why do students withdraw from online graduate nursing and health studies education? J. Educ. 5:n1. doi: 10.9743/JEO.2008.1.2

[ref80] Pierrakeas C. Xeno M. Panagiotakopoulos C. Vergidis D. (2004). A comparative study of dropout rates and causes for two different distance education courses. Int. Rev. Res. Open Dist. Learn. 5:183. doi: 10.19173/irrodl.v5i2.183

[ref81] Pigliapoco E. Bogliolo A. (2008). The effects of psychological sense of community in online and face-to-face academic courses. Int. J. Emerg. Technol. Learn. 3, 60–69. doi: 10.3991/ijet.v3i4.201

[ref82] Pittenger A. Doering A. (2010). Influence of motivational design on completion rates in online self-study pharmacy-content courses. Distance Educ. 31, 275–293. doi: 10.1080/01587919.2010.513953

[ref83] Pocock B. Skinner N. Ichii R. (2009). “Work, life and workplace flexibility,” in The Australian Work and Life Index 2009

[ref84] Poellhuber B. Chomienne M. Karsenti T. (2008). The effect of peer collaboration and collaborative learning on self-efficacy and persistence in a learner-paced continuous intake model. Int. J. E-Learn. Dist. Educ. 22, 41–62.

[ref85] Rahmat T. E. Raza S. Zahid H. Abbas J. Sobri F. A. M. Sidiki S. N. (2022). Nexus between integrating technology readiness 2.0 index and students’e-library services adoption amid the COVID-19 challenges: implications based on the theory of planned behavior. J. Educ. Health Promot. 11:50. doi: 10.4103/jehp.jehp_508_21, PMID: 35372596 PMC8974977

[ref86] Roland N. Frenay M. Boudrenghien G. (2018). Understanding academic persistence through the theory of planned behavior: normative factors under investigation. J. Coll. Stud. Retent. Res. Theory Pract. 20, 215–235. doi: 10.1177/1521025116656632

[ref87] Rotter J. B. (1966). Generalized expectancies for internal versus external control of reinforcement. Psychol. Monogr. Gen. Appl. 80, 1–28. doi: 10.1037/h0092976, PMID: 5340840

[ref88] Seaman J. E. Allen I. E. Seaman J. (2018). Grade Increase: Tracking Distance Education in the United States. Babson Survey Research Group. Available at: https://bayviewanalytics.com/reports/gradeincrease.pdf

[ref89] Shah M. Cheng M. (2019). Exploring factors impacting student engagement in open access courses. Open Learn. J. Open Dist. e-Learn. 34, 187–202. doi: 10.1080/02680513.2018.1508337

[ref05] Shaw M. Burrus S. Ferguson K. (2016). Factors that influence student attrition in online courses. Online Journal of Distance Learning Administration 19, 211–231.

[ref90] Sitzmann T. Kraiger K. Stewart D. Wisher R. (2006). The comparative effectiveness of web-based and classroom instruction: a meta-analysis. Pers. Psychol. 59, 623–664. doi: 10.1111/j.1744-6570.2006.00049.x

[ref91] Stanford-Bowers D. E. (2008). Persistence in online classes: a study of perceptions among community college stakeholders. J. Online Learn. Teach. 4, 37–50.

[ref06] Stewart J. F. Mallery C. Choi J. (2013). College student persistence: a multilevel analysis of distance learning course completion. J. Coll. Stud. Retent.: Res. Theory Pract. 15, 367–385.doi: 10.2190/CS.15.3.d

[ref92] Stone C. O’Shea S. E. (2013). Time, money, leisure and guilt-the gendered challenges of higher education for mature-age students. Austral. J. Adult Learn. 53, 95–116.

[ref93] Strauss A. L. (1987). Qualitative Analysis for Social Scientists, Cambridge, England: University Press.

[ref94] Street H. (2010). Factors influencing a learner’s decision to drop-out or persist in higher education distance learning. Online J. Dist. Learn. Admin. 13.

[ref95] Syverson M. A. Slatin J. (2010). Evaluating learning in virtual environments. http://www.learningrecord.org/caeti.html

[ref96] Tello S. F. (2008). “An analysis of student persistence in online education,” in Information Communication Technologies: Concepts, Methodologies, Tools, and Applications (IGI Global), 1163–1178.

[ref97] Toquero C. M. (2020). Challenges and opportunities for higher education amid the COVID-19 pandemic: the Philippine context. Pedagogical Res. 5:7947. doi: 10.29333/pr/7947

[ref98] Traver A. E. Volchok E. Bidjerano T. Shea P. (2014). Correlating community college students’ perceptions of community of inquiry presences with their completion of blended courses. Internet High. Educ. 20, 1–9. doi: 10.1016/j.iheduc.2013.09.001

[ref99] Willging P. A. Johnson S. D. (2009). Factors that influence students’ decision to dropout of online courses. J. Asynchron. Learn. Netw. 13, 115–127. doi: 10.24059/olj.v13i3.1659

[ref100] Wladis C. Conway K. M. Hachey A. C. (2015). The online STEM classroom—who succeeds? An exploration of the impact of ethnicity, gender, and non-traditional student characteristics in the community college context. Community Coll. Rev. 43, 142–164. doi: 10.1177/0091552115571729

[ref101] Wladis C. Conway K. Hachey A. C. (2017). Using course-level factors as predictors of online course outcomes: a multi-level analysis at a US urban community college. Stud. High. Educ. 42, 184–200. doi: 10.1080/03075079.2015.1045478

[ref102] Wladis C. Hachey A. C. Conway K. (2014). An investigation of course-level factors as predictors of online STEM course outcomes. Comput. Educ. 77, 145–150. doi: 10.1016/j.compedu.2014.04.015

[ref103] Wuellner M. R. (2013). Student learning and instructor investment in online and face-to-face natural resources courses. Nat. Sci. Educ. 42, 14–23. doi: 10.4195/nse.2012.0023

[ref104] Xavier M. Meneses J. (2020). Dropout in Online Higher Education: A Scoping Review From 2014 to 2018. Barcelona: ELearn Center, Universitat Oberta de Catalunya.

[ref105] Xenos M. Pierrakeas C. Pintelas P. (2002). A survey on student dropout rates and dropout causes concerning the students in the course of informatics of the Hellenic open university. Comput. Educ. 39, 361–377. doi: 10.1016/S0360-1315(02)00072-6

[ref106] Xia J. C. Fielder J. Siragusa L. (2013). “Achieving better peer interaction in online discussion forums: a reflective practitioner case study,” in Issues in Educational Research, *Vol*. 23, 97–113.

[ref107] Xu D. Jaggars S. S. (2011). The effectiveness of distance education across Virginia’s community colleges: evidence from introductory college-level math and English courses. Educ. Eval. Policy Anal. 33, 360–377. doi: 10.3102/0162373711413814

[ref108] Yang D. Baldwin S. Snelson C. (2017). Persistence factors revealed: students’ reflections on completing a fully online program. Distance Educ. 38, 23–36. doi: 10.1080/01587919.2017.1299561

[ref109] You J. W. (2018). Testing the three-way interaction effect of academic stress, academic self-efficacy, and task value on persistence in learning among Korean college students. High. Educ. 76, 921–935. doi: 10.1007/s10734-018-0255-0

[ref110] Zimmerman B. J. (2011). “Motivational sources and outcomes of self-regulated learning and performance,” in Handbook of Self-Regulation of Learning and Performance, *Vol*. 5, 49–64.

[ref111] Zimmerman T. D. (2012). Exploring learner to content interaction as a success factor in online courses. Int. Rev. Res. Open Dist. Learn. 13, 152–165. doi: 10.19173/irrodl.v13i4.1302

